# Dietary supplementation with organic choline improves layer performance, egg production, and egg quality

**DOI:** 10.1093/jas/skaf426

**Published:** 2025-12-13

**Authors:** Chia Juan Lim, Jue Ying Wong, Winston Sai Kaw Leow, Lumsangkul Chompunut, Yee Ting Wong

**Affiliations:** Kemin Industries (Asia) Pte Ltd, 758200, Singapore; Kemin Industries (Asia) Pte Ltd, 758200, Singapore; Kemin Industries (Asia) Pte Ltd, 758200, Singapore; Faculty of Agriculture, Chiang Mai University, Chiang Mai 50200, Thailand; Kemin Industries (Asia) Pte Ltd, 758200, Singapore

**Keywords:** choline chloride, choline propionate, egg laying performance, egg quality, fatty liver, laying hen

## Abstract

Choline is vital for liver function and productivity in laying hens, serving as a methyl donor, lipotropic agent, and precursor for key biomolecules like phosphatidylcholine and acetylcholine. Methionine also donates methyl groups and supports protein and feather development. This study investigated choline propionate, a novel organic choline source, versus conventional choline chloride in laying hen diets. A total of 576 Hy-Line Brown hens (50 wk old) were allocated to two trials (288 birds each) with four treatment groups (6 replicates of 12 hens). Trial 1 evaluated choline propionate (120 ppm, 160 ppm) and choline chloride (160 ppm) in a choline-deficient diet. Trial 2 assessed methionine-sparing effects with a 15% methionine and cysteine reduction. Experimental data was analyzed using analysis of variance (ANOVA) procedure of SAS, by the comparison of Least Square Means (LSM) using either Tukey’s parametric test for egg production performance, egg quality parameters, serum biochemical markers, lipids, and fatty acid profiles or Kruskal-Wallis non-parametric test for liver histopathological and liver lesion scoring. In Trial 1, both choline sources improved egg production and quality. Egg production rate with choline propionate supplementation achieved more than 88% compared to 32% in the control group. Overall, significant egg weight increase of more than 3% was recorded for the 120 and 160 ppm choline propionate groups compared to the control. Egg shell strength improved by more than 30% with the choline propionate supplementation compared to the control. Choline propionate at 160 ppm showed significant enhancement of yolk color by 2% (*P* < 0.0001) when compared to the control group. A notable 25% lower serum aminotransferase (AST) levels was observed in the 160 ppm choline propionate group, suggesting a liver protection function compared to the control group. In Trial 2, methionine reduction had no significant effect on performance, with egg production improved numerically in choline-treated groups, suggesting mild methionine-sparing activity. Hens supplemented with choline propionate showed no visible signs of liver steatosis or discoloration, suggestive of improved hepatic condition while controls showed signs of fatty liver traits. Choline propionate demonstrated functional benefits in laying hens under the current study conditions, supporting its relevance as an alternative, supplementary choline source.

## Teaser

Discover how a novel organic choline, choline propionate—outperforms conventional choline chloride in enhancing egg production, liver health, and egg quality in laying hens. This breakthrough offers a practical, and more stable alternative to traditional additives, sparking new possibilities for precise poultry nutrition.

## Introduction

Choline, a quaternary ethanolamine that predominantly exists in the form of phospholipids, plays multiple biological roles in poultry. It is critical for the construction and maintenance of cell membranes and organelles such as mitochondria and microsomes and for the proper development of cartilage matrix in bones (Arele et al., 2015). As a precursor of acetylcholine—the primary neurotransmitter involved in synaptic transmission—it also plays a key role in the nervous system (Wauben and Wainwright, 1999). Choline’s most distinctive physiological role is as a methyl donor, contributing labile methyl groups through its oxidation to betaine, which supports the remethylation of homocysteine to methionine (Halver and Ronald, 2002; Zhang et al., 2013). Furthermore, choline acts as a lipotropic agent, preventing excess fat accumulation in the liver.

While choline can be synthesized de novo, its endogenous production is often insufficient in poultry, leading to deficiency symptoms such as growth retardation and perosis in young chicks. The absorption and utilization of choline vary widely depending on dietary source, strain, age, feed intake, and levels of crude protein and methionine (National Research Council, 1994; Ghazalah, 1998; Workel et al., 2002). Notably, dietary methionine or methyl donors alone may not fully meet choline demands, as avian species have a limited capacity to perform the first step in choline biosynthesis—the methylation of aminoethanol to methyl aminoethanol (Dilger et al., 2007). This is in contrast to mammals like pigs and rats, which readily perform this step (Kroening and Pond, 1967; Baker and Sugahara, 1970). Therefore, dietary supplementation of choline—typically in the form of choline chloride—has become essential in poultry production.

However, choline chloride is highly hygroscopic, leading to handling difficulty and feed stability issues. This limitation has prompted efforts to explore organic or herbal alternatives to choline chloride (D’Souza and Selvam, 2022). Methionine (Met) also serves as a methyl donor and is the first limiting amino acid in corn-soy poultry diets, crucial for protein synthesis, feathering, and growth (Brosnan and Brosnan, 2006; Bunchasak, 2009). Additionally, Met supports redox balance by contributing to glutathione production (Seidel et al., 2019; Wang et al., 2019) and enhances oxidative status under stress conditions (Del Vesco et al., 2014; Magnuson et al., 2020). Because both choline and methionine serve as methyl donors, choline is known to exert a methionine-sparing effect. This effect has been demonstrated in broilers under heat stress, where dietary choline improved feed conversion and reduced serum uric acid levels in methionine-deficient diets (Mahmoudi et al., 2018).

Choline propionate is a novel ionic liquid, consisting of choline cation and propionate anion. It can be synthesized via an acid–base reaction between choline hydroxide and propionic acid (Muhammad et al., 2012) or via ionic exchange involving choline chloride, sodium hydroxide, and propionic acid (Raczek and Mollenkopf, 2002). In this study, the biological efficacy of choline propionate was evaluated for the first time in laying hens, assessing its impact on egg production, egg quality, liver health, and methionine-sparing potential.

The ionic nature of choline propionate enhances its physicochemical stability and solubility, enabling improved bioavailability and biological efficacy upon dietary supplementation. This is supported by prior work demonstrating that choline-carboxylate ionic liquids, form microemulsions and nanostructures that facilitate membrane permeation and epithelial absorption in vivo (Islam et al., 2020; Moshikur et al., 2023). The propionate anion, with short-chain fatty acid has been shown to have metabolic functions, further contributing to hepatic lipid regulation, anti-inflammatory responses, and potential gut microbiota modulation (Santos et al., 2020). In comparison to herbal or natural choline sources (e.g., those derived from soybean lecithin, herbal extracts, or betaine-rich botanicals), which may exhibit batch-to-batch variability, lower choline density, and inconsistent release profiles (D’Souza and Selvam, 2022), choline propionate offers a precise, synthetically defined, and scalable alternative. Herbal choline precursors typically rely on phospholipid-associated choline or betaine for delivery, and their conversion efficiency can vary depending on gut enzymatic activity and intestinal integrity, limiting their functional predictability. Moreover, studies on herbal choline often lack specific molecular mechanistic clarity or in vivo validation of absorption kinetics and methylation equivalence.

Compared to traditional choline chloride, choline propionate demonstrates lower hygroscopicity, reducing formulation and handling issues in feed. In our study, compared to conventional choline chloride, choline propionate demonstrated comparable or superior effects on performance, yolk pigmentation (*P* < 0.0001), liver enzyme activity—aspartate aminotransferase (AST, *P* = 0.0169), and overall liver health. These findings highlight the promise of choline propionate as a novel, bioavailable, and effective choline source for enhancing productivity and health in commercial layer nutrition.

## Material and Methods

### Ethics approval

The Institutional Animal Care and Use Committee of Chiang Mai University, Thailand, approved the animal care and experimental techniques utilized in this study (IACUC code: KW-240722-2).

### Animals, experimental designs, and procedures

The study was conducted in 576 layers of commercial strain (Hyline Brown) at 50 wk old. Birds were allocated randomly into two trials (Trial 1 and Trial 2), with each trial containing 4 treatment groups respectively. All treatment diets were randomly allocated to the birds, with each treatment group having 12 replicates, with 3 cages (4 birds/cage) and a total of 72 birds per treatment. Throughout the 8-wk experiment, the chickens had free access to food and drinking water ad libitum.

### Treatment groups and experimental design

The various treatment groups and experimental design for Trial 1 and Trial 2 are explained in Tables 1 and 2, respectively.

**Table 1.** Experimental design and treatment groups for Trial 1 with choline-deficient basal diet

Treatment group	Description	Explanation
**T1**	Basal diet as Control + 1 kg/t P1	Targeted choline content in this feed was 1100 ppm (mg/kg) which was lower than the normal requirements in order to induce a pronounced effects of choline deficiency in the Control group. No added choline chloride or other choline supplement was included in the T1 diet.
**T2**	Basal diet + 160 ppm (mg/kg) choline chloride + 1 kg/t P1	Total choline content = 1100 ppm (mg/kg) choline (basal level from feed materials) +160 ppm (mg/kg) choline chloride = 1260 ppm (mg/kg) choline. The choline content in T2 matched the choline content in Group T4.
**T3**	Basal diet + 120 ppm (mg/kg) choline from P2 at 0.75 kg/t	Total choline = 1100 ppm (mg/kg) choline (basal level from feed materials) +120 ppm (mg/kg) choline from P2 = 1220 ppm (mg/kg) choline
**T4**	Basal diet + 160 ppm (mg/kg) choline from P2 at 1.0 kg/t	Total choline = 1100 ppm (mg/kg) choline (basal level from feed materials) +160 ppm (mg/kg) choline from P2 = 1260 ppm (mg/kg) choline

**Table 2.** Experimental design and treatment groups for Trial 2 with practical diet with reduced methionine and cysteine content

Treatment group	Description	Explanation
**T5**	Practical diet + 1300 ppm (mg/kg) choline (from feed materials) + 1 kg/t P1	Methionine-deficient diet with a top up of DL-Met in the basal level to avoid severe malnutrition and poor health of layers. This practical diet contained choline but with reduced methionine-cysteine level. Total choline = 1300 ppm (mg/kg) choline
**T6^1^**	Practical diet + 1300 ppm (mg/kg) choline (from feed materials) + 160 ppm (mg/kg) choline chloride + P1 at 1 kg/t	Total choline = 1300 ppm (mg/kg) choline from feed +160 ppm (mg/kg) choline chloride =1460 ppm (mg/kg). Total choline content matched the choline content in Group T8.
**T7^1^**	Practical diet + 1300 ppm (mg/kg) choline (from feed materials) + 120 ppm (mg/kg) choline from P2 at 0.75 kg/t	Total choline = 1300 ppm (mg/kg) choline from feed +120 ppm (mg/kg) choline from P2 = 1420 ppm (mg/kg)
**T8^1^**	Practical diet + 1300 ppm (mg/kg) choline (from feed materials) + 160 ppm (mg/kg) choline from P2 at 1.0 kg/t	Total choline = 1300 ppm (mg/kg) choline from feed +160 ppm (mg/kg) choline from P2 = 1460 ppm (mg/kg)

1 T6, T7, and T8 contained added choline to meet the basic choline requirements and to induce the methionine-sparing effect.

### Experimental diets

The diets contained 15–16% of crude protein. The hens were fed with either the choline or methionine-cysteine depleted diets for the initial 2 wk for acclimatization to the test formulation for Trial 1 and Trial 2, respectively. In Trial 1, a basal corn-soy bean meal with distillers dried grains with solubles (SBM-DDGS) diet was formulated with deficient choline content (with no added choline) and this was used as a Control diet (T1). In Trial 2, a practical diet was given to the birds with the methionine and cysteine levels set at 15% lower than the basal diet in Trial T1.

Two test articles, P1 and P2 used in Trial 1 and Trial 2 respectively were provided by Kemin Industries (Asia) Pte Ltd, Singapore. The test article, P1 (Myco CURB AW Liquid (MCL AW)), did not contain any choline. The other Test article, P2 (AW 40) contained 16% choline in the form of choline propionate. The dosages of 0.75 and 1.0 kg/t are recommended application concentrations used for feed additive with choline and also for feed preservation. A third test article, P3 is in the form of synthetic choline chloride (60% w/w purity).

The test articles, P1, P2, and P3, were added in the control basal and practical diets as shown in the treatment design by mixing them in the mash feed as explained in Tables 3 and 4. Nutrient composition of the experimental diets for Trial 1 and 2 are provided under Tables 5 and 6, respectively.

**Table 3.** Feed ingredients for experimental diet in Trial 1

	Treatment			
Ingredients (% in formula)	T1	T2	T3	T4
**Broken rice**	17.377	17.377	17.377	17.377
**Corn**	39.719	39.719	39.719	39.719
**Rice bran**	10.426	10.426	10.426	10.426
**Soybean meal (44%)**	17.377	17.377	17.377	17.377
**Full fat soybean**	1.490	1.490	1.490	1.490
**DL-Methionine**	0.149	0.149	0.149	0.149
**Calcium carbonate**	4.468	4.468	4.468	4.468
**Tricalcium phosphate 14%**	0.497	0.497	0.497	0.497
**Salt**	0.199	0.199	0.199	0.199
**MAP VITA MIX (Premix)**	0.248	0.248	0.248	0.248
**Meat meal 50%**	2.482	2.482	2.482	2.482
**Microsorb plus (mycotoxin binder)**	0.099	0.099	0.099	0.099
**Limestone**	2.482	2.482	2.482	2.482
**Multi protein plus 68%**	0.993	0.993	0.993	0.993
**Pigment**	0.002	0.002	0.002	0.002
**Phytase**	0.006	0.006	0.006	0.006
**Cow bone**	1.986	1.986	1.986	1.986
**Choline chloride 60% (P3)**	–	0.036	–	–
**MCL AW (P1)**	0.100	0.100	–	–
**AW40 (P2)**	–	–	0.075	0.100

**Table 4.** Feed ingredients for experimental diet in Trial 2

	Treatment			
Ingredients (% in formula)	T5	T6	T7	T8
**Broken rice**	17.377	17.377	17.377	17.377
**Corn**	39.719	39.719	39.719	39.719
**Rice bran**	10.426	10.426	10.426	10.426
**Soybean meal (44%)**	17.377	17.377	17.377	17.377
**Full fat soybean**	1.490	1.490	1.490	1.490
**DL-Methionine**	0.087	0.087	0.087	0.087
**Calcium carbonate**	4.468	4.468	4.468	4.468
**Tricalcium phosphate 14%**	0.497	0.497	0.497	0.497
**Salt**	0.199	0.199	0.199	0.199
**MAP VITA MIX (Premix)**	0.248	0.248	0.248	0.248
**Meat meal 50%**	2.482	2.482	2.482	2.482
**Microsorb plus (mycotoxin binder)**	0.099	0.099	0.099	0.099
**Limestone**	2.482	2.482	2.482	2.482
**Multi protein plus 68%**	0.993	0.993	0.993	0.993
**Pigment**	0.002	0.002	0.002	0.002
**Phytase**	0.006	0.006	0.006	0.006
**Cow bone**	1.986	1.986	1.986	1.986
**Choline chloride 60% (P3)**	0.043	0.079	0.043	0.043
**MCL AW (P1)**	0.100	0.100	–	–
**AW40 (P2)**	–	–	0.075	0.100

**Table 5.** Nutrient composition of experimental diet in Trial 1

	Treatment			
Nutrient composition (%)	T1	T2	T3	T4
**Energy (kcal/kg)**	2771.9	2771.9	2771.9	2771.9
**Protein (%)**	16.32	16.32	16.32	16.32
**Fat (%)**	4	4	4	4
**Crude fiber (%)**	3.55	3.55	3.55	3.55
**Calcium (%)**	3.56	3.56	3.56	3.56
**Total phosphorus (%)**	0.86	0.86	0.86	0.86
**Available phosphorus (%)**	0.54	0.54	0.54	0.54
**Salt (%)**	0.26	0.26	0.26	0.26
**Arginine (%)**	1.05	1.05	1.05	1.05
**Lysine (%)**	0.82	0.82	0.82	0.82
**Methionine + cysteine (%)**	0.64	0.64	0.64	0.64
**Methionine (%)**	0.42	0.42	0.42	0.42
**Threonine (%)**	0.59	0.59	0.59	0.59
**Tryptophan (%)**	0.18	0.18	0.18	0.18
**Linoleic (%)**	1.4	1.4	1.4	1.4
**Choline (mg/kg) (calculated)**	1100	1260	1220	1260
**Choline (mg/kg) (measured)**	859.7^d^	990.8^b^	960.9^c^	1129.5^a^
**Choline recovery, %**	78.2%	78.6%	87.4%	89.6%

**Table 6.** Nutrient composition of experimental diet in Trial 2

	Treatment			
Nutrient composition (%)	T5	T6	T7	T8
**Energy, (kcal/kg)**	2770.5	2770.5	2770.5	2770.5
**Protein (%)**	16.3	16.3	16.3	16.3
**Fat (%)**	4	4	4	4
**Crude fiber (%)**	3.55	3.55	3.55	3.55
**Calcium (%)**	3.56	3.56	3.56	3.56
**Total phosphorus (%)**	0.86	0.86	0.86	0.86
**Available phosphorus (%)**	0.54	0.54	0.54	0.54
**Salt (%)**	0.26	0.26	0.26	0.26
**Arginine (%)**	1.05	1.05	1.05	1.05
**Lysine (%)**	0.82	0.82	0.82	0.82
**Methionine + cysteine (%)**	0.58	0.58	0.58	0.58
**Methionine (%)**	0.36	0.36	0.36	0.36
**Threonine (%)**	0.59	0.59	0.59	0.59
**Tryptophan (%)**	0.18	0.18	0.18	0.18
**Linoleic (%)**	1.4	1.4	1.4	1.4
**Choline (mg/kg) (calculated)**	1300	1460	1420	1460
**Choline (mg/kg) (measured)**	1119.6^d^	1337.9^b^	1310.8^c^	1469.7^a^
**Choline recovery, %**	86.1	91.6	92.3	100.7

### Animal production performance measurement

Layers were fed the experimental diets for a period of 8 wk. The initial feed and residual feed amount of each replicate were recorded everyday divided by the total egg mass (feed/egg mass, g/g). Egg production is expressed as average hen-day production, calculated from the total eggs divided by the total number of days.

### Egg quality analysis

From week 1 to 8, 30 eggs/group were collected each week to analyze for egg quality. Eggshell thickness was measured by using a digital vernier caliper. Eggshell, albumin, and yolk weight were measured using a digital scale. To determine another egg quality, the Digital Egg Tester DET 6000 (NABEL Co., Ltd, Kyoto, Japan) was utilized to evaluate the egg weight, eggshell strength, albumen height, Haugh unit (HU), and yolk color.

### Blood serum collection

Blood samples were collected in vacutainer tubes through the brachial vein puncture. To separate serum, blood was allowed to clot at room temperature, undisturbed for a minimum of 30 minutes. Clot was removed by centrifugation at 1,500 × *g* for 10 min using a refrigerated centrifuge. Following centrifugation, serum was immediately transferred into sterile polypropylene tubes. The samples were maintained on wet ice during handling and stored at –80°C until further analysis.

### Determination of serum aspartate aminotransferase (AST) activity

In accordance with the protocol of an aspartate aminotransferase (AST) Test Kit, the AST activity (IU/L) was determined by the kit using a BioMajesty^®^ JCA-BM6010/C DiaSys Diagnostic Systems (Holzheim, Germany) with the automated Chemistry Analyser BX-301 (Asia Green, Singapore).

### Determination of cholesterol

A piece of breast muscle tissue and abdominal fat (weighing about 250 mg) was homogenized in 2,500 μL of 10 mM phosphate-buffered saline (PBS) and then centrifuged at 1,600 × *g* for 10 minutes at 40°C. The supernatant was used directly in the protocol of a Cholesterol Test Kit (BioMajesty^®^ JCA-BM6010/C: Cholesterol FS) with the automated Chemistry Analyser BX-301 (Asia Green, Singapore). Cholesterol concentration (mg/dL) was calculated.

### Determination of triglycerides

A piece of breast muscle and abdominal fat (weighing about 250 mg) was homogenized in 2,500 μL of 10 mM PBS buffer and centrifuged at 1,600 g for 10 min at 40°C. The supernatant was used directly in the Triglyceride Test Kit (BioMajesty^®^ JCA-BM6010/C: Triglycerides FS) with automated Chemistry Analyser BX-301 (Asia Green, Singapore). Triglyceride concentration (mg/dL) was calculated.

### Total lipids and fatty acids

Eggs and liver of birds were collected at the end of the experiment and was analyzed for Total Lipids and Fatty Acids. Total Lipids of eggs and liver were analyzed by the Soxhlet method using a solvent extraction system (the Soxtec™ 8000, Foss Tecator AB, Sweden).

For fatty acid composition analysis, in accordance with method by (Folch et al., 1957), lipids from the experimental diet, eggs and liver samples were extracted with a chloroform-methanol (2:1) solution. Fatty acids were transformed into fatty acid methyl esters (FAME) using the method described by Morisson and Smith (1964). The fatty acid content was determined using gas chromatography (GC). The gas chromatograph used for the experiment was the ShimadzuGC-2030, manufactured by Shimadzu in Kyoto, Japan. It was fitted with a Restek RT-2560 wall-coated fused wax capillary column, with dimensions of 0.25 mm × 100 m × 0.25 µm. The column was provided by Restek, located in Bellefonte, PA, USA. Helium served as the carrier gas. The temperatures of the injectors were maintained at 250°C. The oven temperature was set to increase at a rate of 3°C per minute, starting at 100°C and reaching 240°C. It then stayed at 240°C for a duration of 20 min. The samples were introduced into the system, and the flame ionization detector was adjusted to a temperature of 250°C. The identity of the samples was determined by comparing the retention durations of their peaks to those of FAME standard mixtures from Restek, located in Bellefonte, PA, USA.

### Fatty liver assessment

After 8 wk of intervention, 12 birds per treatment with body weight close to the pen average were selected and euthanized by a CO_2_ chamber. The liver from each bird was taken for fatty liver assessment (visual scoring).

### Liver histopathology

Liver samples were blotted with tissue paper to remove blood and preserved in 10% neutral buffered formalin. Formalin-fixed liver samples were dehydrated in the series of ascending grades of alcohol (70, 80, 95, and 100%), cleared in chloroform, impregnated with paraffin, and embedded in paraffin wax with ceresin. Paraffin blocks were sectioned at 4 μm thickness using a sliding microtome. After sectioning, the sections were de-paraffinized in xylol followed by hydration in descending grades of alcohol (100, 95, 80, and 70%) and distilled water. The sections were stained with standard hematoxylin and eosin (H&E) method and then mounted (DPX mountant) on microscope slides for viewing and assessment.

### Choline content and choline recovery in diets

#### Sample preparation

Homogenized powdered mash feed samples (20 g) were extracted with 100 mL of methanol and mixed for 5 min. The mixture was transferred to a 250 mL volumetric flask and top up with methanol. The mixture was thoroughly mixed and then filtered thought a Whatman No. 1 filter paper. A 50 mL aliquot was taken out and the solvent was dried by evaporation. The residue was dissolved with 50 ml deionized (DI) water containing 0.5 g calcium hydroxide and then filtered by a 0.45 μm filter membrane. Samples were analyzed using a capillary electrophoresis equipment.


Percent choline % recovery = [choline in feed (mg/g sample)/Total amount of choline content in the diet] × 100%

### Capillary electrophoresis analysis

Experimental conditions and parameters for capillary electrophoresis analysis were performed in a previously published method (Tran et al., 2022). The choline quantification method was previously validated with the limits of detection (signal-to-noise ratio = 3) and quantitation (signal-to-noise ratio = 10) were 14.7 and 48.9 mg L^−1^, respectively. The peak area and migration time intraday and interday precision (percent RSD) were all <15%, and the recoveries ranged from 79.4 to 115.2% at different spiking levels. The linearity over 100–1,000 ppm (*R*^2^ ≈ 0.994) indicated good calibration behavior in this working range (Tran et al., 2022). Briefly, a Capillary Electrophoresis equipment (Brand: Capel-205, Lumex Instruments) was used. Mobile phase eluent contained a mixture of Benzimidazole and Tartaric acid, 18-Crown-6 Solution and deionized water. The analytical parameters were as follows:

**Table:** 

**Wavelength, nm**	276
**Inject**	30 mbar, 5 s
**Voltage, kV**	+ 25
**Temperature, °C**	20
**Analysis time, min**	8
**Function**	Indirect (Invert)

### Statistical analysis

All experimental data was analyzed using analysis of variance (ANOVA) procedure of SAS Enterprise Guide Software V.9.4 (SAS Institute, Cary, NC, USA), by the comparison of Least Square Means (LSM) using either Tukey’s parametric test (for egg production performance (hen-day egg production (%), Average daily feed intake (ADFI), egg laying feed conversion ratio (FCR), average egg mass, etc); egg quality parameters (Haugh unit, yolk color score, shell thickness, etc), serum biochemical markers (AST, ALT, total cholesterol, triglycerides) and egg and liver lipids and fatty acid profiles (total lipids, PUFA, etc.)) or Kruskal-Wallis non-parametric test (for liver histopathological and liver lesion scoring). Statistical significance was determined when the two-tailed probability, due to random variation, was less than 5% (*P* < 0.05).

## Results

### Results for Trial 1

#### Animal production performance

Production performance from hens fed experimental diets is shown in Table 7. The result showed that hens fed with choline-deficient diet (Control T1) had significantly reduced egg production (>169% reduction), egg mass (>235% reduction), and average daily feed intake (ADFI) (>19% reduction) (*P* < 0.0001), as well as higher in feed conversion ratio (FCR) (higher by > 2.9 points) (*P* < 0.005) compared to treatments groups with choline supplementation (T2, T3, T4). Moreover, the addition of the new choline propionate formulation (T3 and T4) resulted in improvement in egg production capacity with an increase of 177 and 181%, respectively. Overall egg mass also increased by 245 and 248%, with a decrease in feed conversion ratio (reduction by 2.89 and 2.94 points) for the choline propionate groups, T3 and T4, respectively, when compared to the Control (T1) group.

**Table 7.** Effect of choline product supplementation on productive performance of laying hens in Trial 1

	Treatment					
Item	T1	T2	T3	T4	SEM	*P*-value
***1–4 wk of experiment***						
**Egg production (%)**	49.2^b^	87.7^a^	88.9^a^	89.9^a^	1.805	<0.0001
**ADFI (g)**	105.6^b^	117.4^a^	117.2^a^	118.1^a^	1.009	<0.0001
**Egg mass (g)**	29.9^b^	56.6^a^	56.6^a^	56.9^a^	1.348	<0.0001
**FCR, feed/egg mass, g/g**	3.03^a^	2.09^b^	2.19^b^	2.09^b^	0.194	0.0063
***5–8 wk of experiment***						
**Egg production (%)**	17.8^c^	83.9^b^	87.7^a^	89.0^a^	0.932	<0.0001
**ADFI (g)**	93.8^b^	119.6^a^	119.0^a^	119.3^a^	1.104	<0.0001
**Egg mass (g)**	2.4^c^	51.8^b^	54.5^a^	55.3^a^	0.847	<0.0001
**FCR, feed/egg mass, g/g**	9.79^a^	2.31^b^	2.18^b^	2.17^b^	1.270	0.0014
***1–8 wk of experiment***						
**Egg production (%)**	31.8^c^	85.6^b^	88.3^a,b^	89.4^a^	1.087	<0.0001
**ADFI (g)**	99.1^b^	118.6^a^	118.2^a^	118.8^a^	0.805	<0.0001
**Egg mass (g)**	16.1^b^	54.2^a^	55.6^a^	56.1^a^	0.948	<0.0001
**FCR, feed/egg mass, g/g**	5.07^a^	2.21^b^	2.18^b^	2.13^b^	0.528	0.0014

a,b Means within the same row with different letter differ significantly (*P* < 0.05).

#### Egg quality

During 8 wk of feeding, the choline-deficient diet (Control T1) negatively affected the egg quality including egg weight, eggshell weight, eggshell breaking strength, yolk weight, eggshell thickness, and eggshell strength of hens (Table 8). Egg weight improved significantly by >3% with choline supplementation compared to the Control T1 group. Notably, supplementation with the new choline propionate (T3 and T4) enhanced the egg quality in terms of egg shell strength (>29% increase) and shell thickness (>38% increase) compared to the T1 Control hens and the results were comparable to the choline chloride treatment group (T2). Furthermore, yolk weight was significantly increased (>3%) with the choline propionate (T3 and T4) treatments compared to T1 Control and T2 choline chloride groups. Interestingly, treatment group T4 with choline propionate showed highest yolk color fan score with an increase of 0.21 and 0.32 point (*P* < 0.0001) compared to the T1 Control and T2 groups, respectively.

**Table 8.** Effect of choline product supplementation on egg quality in Trial 1

	Treatment					
Item	T1	T2	T3	T4	SEM	*P*-value
***1–4 wk of experiment***						
**Egg weight (g)**	61.0	61.9	61.6	60.7	0.560	0.4129
**Egg shell weight (g)**	6.81^b^	8.03^a^	7.87^a^	7.85^a^	0.109	<0.0001
**Yolk weight (g)**	13.19^b^	13.28^b^	13.99^a^	13.83^a,b^	0.218	0.0431
**Albumin weight (g)**	37.09	38.95	38.12	37.35	0.524	0.0843
**Strength (Kgf)**	2.56^b^	5.01^a^	4.85^a^	4.97^a^	0.134	<0.0001
**Shell-thickness (mm)**	0.232^b^	0.334^a^	0.328^a^	0.330^a^	0.005	<0.0001
**Albumin height**	8.02	7.68	7.72	7.57	0.148	0.1963
**Haugh unit**	88.25	86.90	87.01	86.46	0.938	0.5795
**Yolk color fan score**	10.59^a,b^	10.28^b^	10.48^c^	10.75^a^	0.059	0.0001
***5–8 wk of experiment***						
**Egg weight (g)**	58.2^b^	61.9^a^	63.2^a^	62.2^a^	0.526	<0.0001
**Egg shell weight (g)**	6.77^b^	9.04^a^	8.94^a^	9.00^a^	0.112	<0.0001
**Yolk weight (g)**	16.21^a^	15.56^b^	16.29^a^	16.04^a^	0.139	0.0068
**Albumin weight (g)**	24.67^b^	37.30^a^	37.98^a^	37.17^a^	1.102	<0.0001
**Strength (Kgf)**	7.37	7.59	7.63	7.58	0.114	0.3892
**Shell-thinkness (mm)**	0.218^b^	0.300^a^	0.295^a^	0.303^a^	0.004	<0.0001
**Albumin height**	9.74	9.46	9.60	9.71	0.112	0.3106
**Haugh unit**	86.13^a,b^	87.25^a^	86.03^a,b^	84.86^b^	0.709	0.161
**Yolk color fan score**	10.37^b^	10.40^b^	10.46^b^	10.61^a^	0.052	0.0172
***1–8 wk of experiment***						
**Egg weight (g)**	59.7^b^	61.9^a^	62.5^a^	61.5^a^	0.418	0.001
**Egg shell weight (g)**	6.83^b^	8.59^a^	8.47^a,b^	8.49^a^	0.078	<0.0001
**Yolk weight (g)**	14.68^b^	14.55^b^	15.27^a^	15.06^a^	0.122	0.0016
**Albumin weight (g)**	30.57^b^	38.04^a^	38.04^a^	37.25^a^	0.599	<0.0001
**Eggshell strength (kgf)**	4.93^b^	6.45^a^	6.39^a^	6.42^a^	0.137	<0.0001
**Shell-thickness (mm)**	0.225^b^	0.315^a^	0.310^a^	0.315^a^	0.003	<0.0001
**Albumin height**	8.82	8.67	8.76	8.76	0.078	0.6119
**Haugh unit**	87.12	87.10	86.47	85.57	0.551	0.1913
**Yolk color fan score**	10.46^b^	10.35^b^	10.47^b^	10.67^a^	0.039	0.0001

a,b Means within the same row with different superscripts differ significantly (*P* < 0.05).

#### Blood serum biomarkers

Serum aspartate aminotransferase (AST) activity of treatment groups in Trial 1 was measured (Table 9). It was found that the serum AST level in hens fed with at choline propionate product at 1.0 kg/t (T4) was significantly lower than the hens fed with choline-deficient diet (T1) and other choline-supplemented diets (T2 and T3) (*P *= 0.0149).

**Table 9.** Effect of choline product supplementation on serum AST, cholesterol, and triglyceride content in breast muscle and abdominal fat of laying hens in Trial 1

	Treatment					
Item	T1	T2	T3	T4	SEM	*P*-value
***Serum***						
**AST, U/L**	212^a^	239^a^	199^a,b^	160^b^	14.5	0.0169
***Breast muscle***						
**Cholesterol, mg/dL**	6.5^a^	2.5^b^	3.5^b^	2.0^b^	0.92	0.0208
**Triglyceride, mg/dL**	29.6^a^	15.5^b^	19.3^b^	14.8^b^	2.26	0.0019
***Abdominal fat***						
**Cholesterol, mg/dL**	1.50	2.25	1.50	1.75	0.27	0.2170
**Triglyceride, mg/dL**	72.3	89.0	69.5	63.5	9.24	0.2917

a,b Means within the same row with different superscripts differ significantly (*P* < 0.05).

#### Cholesterol and triglyceride in breast muscle and abdominal fat

Cholesterol and triglyceride content in breast muscle and abdominal fat of treatment group T1 to T4 is shown in Table 9. Treatment groups with choline supplementation (T2, T3, T4) showed significantly lower level of cholesterol (*P = *0.0208) and triglycerides (*P *= 0.0019) in the breast muscle compared to Control T1. However, there was no significant difference in the abdominal fat content among the treatment groups.

#### Total lipids in eggs and livers

Total lipids in the eggs for groups T1 to T4 did not show any significant difference among them. However, the total lipids found in liver for the choline treatment groups (T2, T3, T4) were numerically lower (>18% reduction) than in Control (T1) as shown in Table 10 although not statistically different.

**Table 10.** Effect of choline product supplementation on total lipids in egg and liver from laying hens in Trial 1

	Treatment					
Item	T1	T2	T3	T4	SEM	*P*-value
**Total lipids (g/100 g)**						
**Egg**	61.31^a^	57.34^a^	51.43^b^	61.30^a^	1.058	0.0263
**Liver**	23.84	18.11	18.24	19.35	1.108	0.1609

a,b Means within the same row with different superscripts differ significantly (*P* < 0.05).

#### Fatty acids in eggs and livers

The analysis of the fatty acids (FAs) (%) profile of eggs was conducted and results are provided in Table 11. The saturated FAs (SFA), palmitic (C16:0), stearic acid (C18:0), as well as in Σ SFA (*P* = 0.0003) of hen fed with the new choline propionate formulation at 0.75 kg/t (T3) was lowest in the hens fed with choline-deficient diet. There was no difference in the total monounsaturated FAs (MUFA), in palmitoleic (C16:1) as well as oleic (C18:1 n9c) among the groups. However, the polyunsaturated FAs (PUFA) presents as ΣPUFA was significantly increased in hen fed with choline propionate product at 0.75 kg/t (T3) compared to the choline chloride group (T2). The omega-3 fatty acids, docosahexaenoic acid (DHA; C22:6 n3), was highest (0.90%) in hens in Group T2 choline chloride compared to the other groups. Total PUFA was highest in T3 (15.3%), followed by the control (13.8%), whereas T4 (10.6%) exhibited the lowest value (*P* < 0.001). Within the PUFA group, linoleic acid (C18:2 n6c) was elevated in T3 (14.3%) but suppressed in T4 (9.6%), and docosahexaenoic acid (DHA, C22:6 n3) was highest in T2 (0.90%), representing a 23% increase over the T1 Control and >25% higher than T3 or T4 (*P* = 0.0017). This enrichment was accompanied by a favorable n‑3/n‑6 ratio in both T2 (6.35) and T4 (6.25), compared with the control (4.24; P = 0.0153). The Σ n-3/n-6 PUFA ratio in the yolks was highest for T2 and T4 groups at 6.35 and 6.25, respectively, compared to the Control T1.

**Table 11.** Effect of choline product supplementation on fatty acid composition of eggs from laying hens in Trial 1

	Treatment					
Total fatty acids, %	T1	T2	T3	T4	SEM	*P*-value
**C14:0**	0.36	0.36	0.33	0.37	0.013	0.1006
**C16:0**	30.5^a,b^	32.7^a^	29.7^b^	31.9^a,b^	0.611	0.0179
**C16:1**	2.20	3.13	2.21	2.87	0.291	0.0965
**C18:0**	7.45	7.12	6.73	7.38	0.243	0.2039
**C18:1 n9c**	43.1	41.4	43.4	44.7	1.040	0.2188
**C18:2 n6c**	12.8^a,b^	11.8^b^	14.3^a^	9.6^c^	0.546	0.0005
**C18:3 n6**	0.06^a^	0.04^b^	0.06^a,b^	0.03^c^	0.005	0.0022
**C18:3 n3**	0.20	0.20	0.20	0.22	0.010	0.2771
**C21:0**	0.17	0.17	0.18	0.16	0.021	0.8880
**C20:2**	0.11	0.09	0.11	0.12	0.009	0.2984
**C20:3 n6**	0.16	0.13	0.14	0.13	0.010	0.0939
**C23:0**	2.22^a^	1.97^b^	1.95^b^	1.69^c^	0.067	0.0012
**C22:6 n3**	0.73^b^	0.90^a^	0.71^b^	0.71^b^	0.030	0.0017
**ΣSFA**	38.3^b^	40.2^a^	36.8^c^	39.7^a^	0.408	0.0003
**ΣMUFA**	45.2	44.6	45.6	47.6	0.847	0.1207
**ΣPUFA**	13.8^a,b^	12.9^b^	15.3^a^	10.6^c^	0.552	0.0005
**SFA/USFA**	0.65^b^	0.70^a^	0.60^c^	0.68^a,b^	0.011	0.0004
**PUFA/MUFA**	0.31^a^	0.29^a^	0.33^a^	0.22^b^	0.017	0.0050
**SFA/PUFA**	2.77^b,c^	3.14^c^	2.41^b^	3.78^a^	0.140	0.0001
**SFA/MUFA**	0.85	0.90	0.81	0.84	0.023	0.0781
**n-3**	0.93^b^	1.09^a^	0.91^b^	0.93^b^	0.037	0.0111
**n-6**	0.23^a^	0.18^b^	0.20^a,b^	0.16^b^	0.015	0.0392
**n-3/n-6**	4.24^b^	6.35^a^	4.58^b^	6.25^a^	0.481	0.0153
**n-6/n-3**	0.25^a^	0.16^b^	0.22^a,b^	0.17^b^	0.019	0.0222

a,b Means within the same row with different superscripts differ significantly (*P* < 0.05).

The results of the FAs content (%) of the liver is shown in Table 12. The saturated FAs (SFA), myristic acid (C14:0), stearic acid (C18:0), as well as in Σ SFA (*P* = 0.0263) with the choline propionate product at 0.75 kg/t (T3) and 1.0 kg/t (T4) was lower compared to the Control T1 group in the choline-deficient diet. There was significantly higher total monounsaturated FAs (MUFA), palmitoleic (C16:1), oleic (C18:1 n9c) as well as Σ MUFA, in hens fed with choline propionate product at 1.0 kg/t (T4) compared to the other groups. The polyunsaturated FAs (PUFA) presented as Σ PUFA was significantly increased in hens fed with choline in T2 and T3 groups compared to the Control group T1. However, there was no statistically difference in the main omega-3 fatty acids, alpha-linolenic acid (ALA; C18:3 n3), docosahexaenoic acid (DHA; C22:6 n3), and Σ *n*−6/P *n*−3 PUFA ratio among all the groups.

**Table 12.** Effect of choline product supplementation on fatty acid composition of liver from laying hens in Trial 1

	Treatment					
Total fatty acids, %	T1	T2	T3	T4	SEM	*P*-value
**C14:0**	0.42^a^	0.38^a^	0.20^b^	0.48^a^	0.041	0.0022
**C16:0**	34.3^b^	35.4^b^	35.3^b^	35.4^a^	0.194	0.0345
**C16:1**	0.97^b^	1.15^b^	0.9^b^	1.76^a^	0.194	0.0345
**C18:0**	14.41^a,b^	16.48^a^	15.83^a^	12.16^b^	0.877	0.0207
**C18:1 n9c**	41.79^a,b^	35.76^b^	37.25^b^	45.13^a^	2.012	0.024
**C18:3 n6**	0.12	0.08	0.16	0.08	0.027	0.1756
**C18:3 n3**	0.19	0.20	0.13	0.15	0.047	0.6592
**C21:0**	0.14	0.22	0.13	0.16	0.051	0.6149
**C23:0**	5.76^b^	7.88^a^	7.78^a^	6.56^a,b^	0.474	0.0234
**C22:6 n3**	1.90	2.49	2.34	1.16	0.341	0.0706
**ΣSFA**	49.12^a,b^	52.23^a^	51.31^a,b^	48.00^b^	0.926	0.0263
**ΣMUFA**	42.75^a,b^	36.91^b^	38.16^b^	46.89^a^	2.204	0.0283
**ΣPUFA**	2.2	2.76	2.62	1.39	0.360	0.0766
**SFA/USFA**	1.10^b,c^	1.32^a^	1.26^a,b^	1.01^c^	0.062	0.0155
**PUFA/MUFA**	0.05^a,b^	0.08^a^	0.07^a^	0.03^b^	0.010	0.0412
**SFA/PUFA**	22.5	19.5	20.2	67.8	13.820	0.0780
**SFA/MUFA**	1.16^b,c^	1.42^a^	1.35^a,b^	1.05^c^	0.074	0.0158
**n-3**	2.10	2.68	2.46	1.31	0.341	0.0668
**n-6**	0.12	0.08	0.16	0.08	0.027	0.1756
**n-3/n-6**	18.6	30.6	17.4	14.5	4.510	0.1691
**n-6/n-3**	0.06	0.03	0.06	0.10	0.020	0.0862

a,b Means within the same row with different superscripts differ significantly (*P* < 0.05).

#### Fatty liver

Table 13 shows the different liver conditions of the laying hens with and without choline product supplementation. The results clearly demonstrated that the highest percentage of the fatty liver incidence was found in the Control T1 hens without choline supplementation (T1) when compared to the other choline-treated groups.

**Table 13.** Incidence of fatty liver in laying hens in Trial 1

Item	Treatment			
	T1	T2	T3	T4
**No. of sample observed**	108	108	108	108
**Rate for the fatty liver incidence (%)**	29.63^a^	16.67^b^	24.07^b^	22.22^b^

a,b Means within the same row with different superscripts differ significantly (*P* < 0.05).

The absence or incidence of fatty liver lesions in laying hens is presented in Table 14. The findings indicated an evident rise in liver inflammation, as evidenced by the presence of a greater number of inflammatory foci, in hens fed a choline-deficient diet (Control T1) compared to the other choline-treated groups. Nevertheless, hens that received the new choline propionate product at 1.0 kg/t (T4) exhibited the least inflammatory foci *P* = 0.05).

**Table 14.** Effect of choline product supplementation on fatty liver lesion severity using histopathology scoring with H&E staining in Trial 1

Lesions	Treatment				
	T1	T2	T3	T4	*P*-value
***Liver***					
**Microvesicular**	0.5 (2-0)	0 (0-0)	0 (1-0)	1.5 (2-0)	0.06
**Macrovesicular steatosis**	0 (1-0)	0 (0-0)	0 (1-0)	0 (2-0)	0.07
**Hypertrophy**	0 (0.75-0)	0 (1-0)	0 (0.75-0)	1 (2-0)	0.15
**Ballooning**	0 (0-0)	0 (0-0)	0 (0-0)	0 (0-0)	0.39
**Fibrosis**	0 (0-0)	0 (0-0)	0 (0-0)	0 (0-0)	0.39
***Inflammation***					
**Number of inflammatory foci**	1^a^ (1-0.25)	0.5^a,b^ (1-0)	0.5^a,b^ (1-0)	0^b^ (0-0)	0.05
**Microgranulomas**	0 (0-0)	0 (0-0)	0 (0-0)	0 (0-0)	0.39
**Large lipogranuloma**	0 (0-0)	0 (0-0)	0 (0-0)	0 (0-0)	0.29
**Portal inflammation**	0.5 (1-0)	0 (1-0)	0.5 (1-0)	0 (0-0)	0.25

a,b Median within the same row with different superscripts differ by Kruskal-Wallis test (*P* < 0.05).

**Figure 1.**
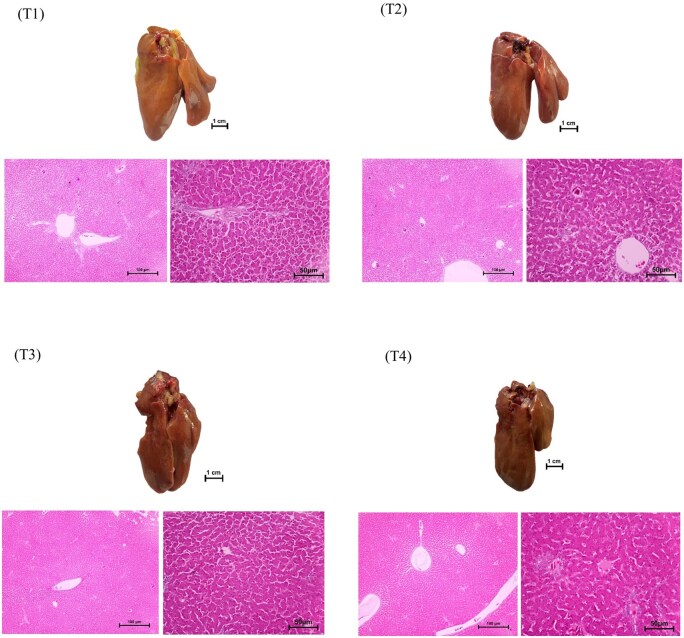
Microscope images illustrating the typical features of liver hepatocytes in terms of gross appearance and sections stained with hematoxylin and eosin (H&E) in laying hens in Trial 1. Picture in T1 showed yellow patches of fatty liver incidence. Pictures in T2, T3, and T4 showed healthy, normal red liver.

Liver histopathology results provided in Figure 1, showed that liver samples from choline-treated groups (T2, T3, T4) showed healthy and normal red tissues which is in contrast from the Control group (T1). Liver samples from the Control T1 group indicated a clear sign of fatty liver with yellow patches of lipid accumulation.

### Results for Trial 2

#### Animal production performance

In Trial 2, there were no significant difference in the layer performance in terms of egg production, egg mass, and average daily feed intake in hens fed with diet with a reduction of 15% methionine and cysteine (Table 15). However, the group treated with choline propionate product at 0.75 kg/t (T7) and 1.0 kg/t diet (T8) resulted in a numerical increase in egg mass when compared to the Control (T5) group and the choline chloride treated Group (T6).

**Table 15.** Effect of choline product supplementation on productive performance of laying hens in Trial 2

	Treatment					
Item	T5	T6	T7	T8	SEM	*P*-value
***1–4 wk of experiment***						
**Egg production (%)**	80.1	85.4	88.3	89.6	3.093	0.1658
**ADFI (g)**	116.2	117.5	118.2	118.0	0.668	0.1824
**Egg mass (g)**	51.6	51.4	56.9	57.3	1.955	0.0675
**FCR, feed/egg mass, g/g**	2.27	2.21	2.10	2.07	0.071	0.1909
***5–8 wk of experiment***						
**Egg production (%)**	80.4	85.0	83.5	86.0	3.708	0.7259
**ADFI (g)**	118.4	118.7	119.1	119.2	0.631	0.7817
**Egg mass (g)**	50.3	53.6	53.6	54.1	2.458	0.6831
**FCR, feed/egg mass, g/g**	2.38	2.24	2.25	2.28	0.112	0.8201
***1–8 wk of experiment***						
**Egg production (%)**	80.2	85.2	85.6	87.6	3.333	0.4638
**ADFI (g)**	117.4	118.2	118.7	118.7	0.617	0.4318
**Egg mass (g)**	51.0	52.5	55.3	55.7	2.035	0.3185
**FCR, feed/egg mass, g/g**	2.33	2.23	2.18	2.19	0.090	0.6395

#### Egg quality

In the overall 8-wk treatment, there was no significant difference in egg quality parameters for all the groups (Table 16). Interestingly, the choline propionate treated groups at 0.75 kg/t (T7) and 1.0 kg/t diets (T8) consistently demonstrated numerically higher values in egg weight, eggshell weight, albumin height, Haugh Unit and yolk color fan score, compared to the Control (T5) and the choline chloride treated Group (T6).

**Table 16.** Effect of choline product supplementation on egg quality in Trial 2

	Treatment					
Item	T5	T6	T7	T8	SEM	*P*-value
***1–4 wk of experiment***						
**Egg weight (g)**	61.4	60.7	62.2	61.5	0.609	0.4542
**Egg shell weight (g)**	7.81	7.82	7.94	7.93	0.107	0.7540
**Yolk weight (g)**	13.92	13.99	13.87	13.94	0.115	0.9104
**Albumin weight (g)**	38.01	37.32	38.76	38.04	0.482	0.2521
**Egg shell strength (kgf)**	4.81	4.77	4.84	4.68	0.122	0.8134
**Shell-thickness (mm)**	0.332	0.337	0.343	0.328	0.006	0.2781
**Albumin height**	7.42	7.40	7.74	7.76	0.135	0.1238
**Haugh unit**	84.96	85.13	86.96	87.07	0.752	0.1074
**Yolk color fan score**	10.76^a^	10.45^b^	10.58^a,b^	10.75^a^	0.063	0.0062
***5-8 wk of experiment***						
**Egg weight (g)**	62.7	62.4	62.9	63.0	0.549	0.8535
**Egg shell weight (g)**	8.76	8.64	8.80	8.91	0.115	0.4342
**Yolk weight (g)**	16.29	16.27	16.19	16.24	0.122	0.9420
**Albumin weight (g)**	37.65	37.49	37.94	37.88	0.444	0.8815
**Strength (kgf)**	7.09^a,b^	7.01^b^	6.93^b^	7.32^a^	0.090	0.0344
**Shell-thickness (mm)**	0.304	0.310	0.303	0.301	0.002	0.0755
**Albumin height**	9.66	9.57	9.68	9.70	0.085	0.6921
**Haugh Unit**	80.84	81.32	80.89	83.55	1.079	0.2674
**Yolk color fan score**	10.48	10.56	10.52	10.52	0.055	0.7679
***1–8 wk of experiment***						
**Egg weight (g)**	62.1	61.7	62.6	62.3	0.504	0.6095
**Egg shell weight (g)**	8.34	8.28	8.42	8.47	0.099	0.5162
**Yolk weight (g)**	15.24	15.25	15.16	15.21	0.101	0.9189
**Albumin weight (g)**	37.81	37.42	38.31	37.95	0.400	0.4844
**Eggshell strength (kgf)**	6.08	6.02	6.00	6.15	0.072	0.4834
**Shell-thickness (mm)**	0.317	0.322	0.321	0.313	0.003	0.2614
**Albumin height**	8.67	8.60	8.82	8.84	0.087	0.1865
**Haugh unit**	82.67	83.01	83.59	85.14	0.734	0.1175
**Yolk color**	10.60	10.51	10.55	10.63	0.044	0.2691

a,b Means within the same row with different superscripts differ significantly (*P* < 0.05).

#### Blood serum biomarkers

Serum aspartate aminotransferase (AST) activity from hens fed experimental diets in Trial 2 showed no significant difference among the treatment groups (Table 17). The abdominal fat cholesterol and triglyceride contents were consistently lower in the choline treated groups (T6, T7, T8) compared to the Control group (T5).

**Table 17.** Effect of choline product supplementation on serum AST, cholesterol, and triglyceride content in breast muscle and abdominal fat of laying hens in Trial 2

	Treatment					
Item	T5	T6	T7	T8	SEM	*P*-value
***Serum***						
**AST, U/L**	159	198	186	187	16.0	0.4105
***Breast muscle***						
**Cholesterol, mg/dL**	2.50	2.00	2.25	3.50	0.37	0.0687
**Triglyceride, mg/dL**	15.6	14.0	16.3	17.0	1.01	0.2466
***Abdominal fat***						
**Cholesterol, mg/dL**	4.50	1.00	1.25	1.00	1.59	0.3666
**Triglyceride, mg/dL**	100.6	52.5	67.5	50.8	22.4	0.4012

#### Total lipids in eggs and livers

Total lipids in egg and in liver from hens fed with experimental diet in Trial 2 is shown in Table 18. The result did not demonstrate significant difference among the groups. As expected, the hens fed with choline supplement (T5, T7, T8) showed numerically lower lipid content in livers compared to the Control Group (T5).

**Table 18.** Effect of choline product supplementation on total lipids in egg and liver from laying hens in Trial 2

	Treatment					
Item	T5	T6	T7	T8	SEM	*P*-value
**Total lipids (g/100 g)**						
**Egg**	63.68	61.00	66.97	60.59	1.047	0.0645
**Liver**	24.34	22.84	20.74	19.97	1.298	0.4220

#### Fatty acids in eggs and livers

Fatty acid profiling in eggs was determined and tabulated in Table 19. The result showed that the total saturated FAs (SFA), the Σ SFA, was highest in the eggs of hen fed with choline propionate product at 1 kg/t (T8). However, for other fatty acid profiles, no obvious trend was observed among the treatment groups. There was significantly higher total monounsaturated FAs (MUFA), oleic (C18:1 n9c) as well as Σ MUFA, in hen fed with choline propionate product at 0.75 kg/t (T7) and 1 kg/t (T8), respectively, compared to the Control (T5) and choline chloride (T6) group. The polyunsaturated FAs (PUFA) presented as Σ PUFA was significantly increased in hen fed with choline chloride (T6) and choline propionate (T8) compared to the Control group (T5). The omega-3 fatty acids, docosahexaenoic acid (DHA; C22:6 n3), was highest (0.76%) in the hens fed with the choline chloride (T6) diet compared to other groups. In addition, the Σ n-3 and Σ n-3/n-6 PUFA ratio was also highest in the hen fed with the diet of T6 compared to other groups. The Σ n-6 and Σ n-6/n-3 PUFA ratio in the yolks was highest in the hens fed with the choline propionate diet (T8) compared to other groups.

**Table 19.** Effect of choline product supplementation on fatty acid composition of eggs from laying hens in Trial 2

	Treatment					
Total fatty acids, %	T5	T6	T7	T8	SEM	*P*-value
**C14:0**	0.40	0.36	0.34	0.35	0.019	0.2070
**C16:0**	30.4	30.9	29.9	31.5	0.384	0.0601
**C16:1**	2.79	2.81	2.85	2.99	0.247	0.9346
**C18:0**	7.54	7.35	7.30	7.59	0.219	0.7372
**C18:1 n9c**	46.3^b^	46.4^b^	49.2^a^	45.7^b^	0.397	0.0002
**C18:2 n6c**	9.7^a^	9.0^a^	7.7^b^	9.1^a^	0.233	0.0004
**C18:3 n6**	0.047^a^	0.035^a,b^	0.025^b^	0.032^b^	0.003	0.0041
**C18:3 n3**	0.17^b^	0.20^a,b^	0.24^a^	0.21^a,b^	0.011	0.0055
**C21:0**	0.11	0.15	0.10	0.09	0.014	0.0698
**C20:2**	0.08	0.15	0.08	0.06	0.021	0.0587
**C20:3 n6**	0.11^a,b^	0.11^a,b^	0.09^b^	0.14^a^	0.010	0.0317
**C23:0**	1.79	1.75	1.69	1.66	0.043	0.1675
**C22:6 n3**	0.60^b^	0.76^a^	0.59^b^	0.58^b^	0.034	0.0074
**ΣSFA**	38.3^a,b^	38.6^a,b^	37.5^b^	39.5^a^	0.380	0.0273
**ΣMUFA**	49.1^b^	49.2^b^	51.9^a^	48.7^b^	0.226	<0.0001
**ΣPUFA**	10.5^a^	10.0^a^	8.51^b^	9.94^a^	0.235	0.0004
**SFA/USFA**	0.64^a,b^	0.65^a,b^	0.62^b^	0.67^a^	0.011	0.039
**PUFA/MUFA**	0.21^a^	0.20^a^	0.16^b^	0.20^a^	0.005	<0.0001
**SFA/PUFA**	3.66^b^	3.86^b^	4.43^a^	3.98^b^	0.136	0.0111
**SFA/MUFA**	0.78^a^	0.78^a^	0.72^b^	0.81^a^	0.011	0.0012
**n-3**	0.77^b^	0.96^a^	0.83^b^	0.79^b^	0.037	0.0148
**n-6**	0.16^a^	0.14^a,b^	0.12^b^	0.17^a^	0.010	0.0155
**n-3/n-6**	4.91^b^	6.87^a^	7.03^a^	4.76^b^	0.587	0.0272
**n-6/n-3**	0.21^a,b^	0.15^b^	0.14^b^	0.22^a^	0.017	0.0145

a,b Means within the same row with different superscripts differ significantly (*P* < 0.05).

The results of the FA content (%) of liver in Table 20 showed no statistical difference in the Σ SFA, Σ MUFA, and Σ PUFA among the groups. However, the result of γ-linolenic acid (C18:3 n6), Σ n-6, and Σ n-6/P n-3 PUFA ratio were significantly highest in the hens fed with choline propionate product at 1.0 kg/t (T8) when compared to the Control group (T5).

**Table 20.** Effect of choline product supplementation on fatty acid composition of livers from laying hens in Trial 2

	Treatment					
Total fatty acids, %	T5	T6	T7	T8	SEM	*P*-value
**C14:0**	0.36	0.37	0.29	0.41	0.041	0.3024
**C16:0**	38.9^a^	33.6^b^	33.8^b^	37.5^a,b^	0.150	0.0271
**C16:1**	1.59^a^	0.97^b^	0.88^b^	1.18^a,b^	0.150	0.0271
**C18:0**	15.42	13.60	15.61	14.41	2.511	0.9349
**C18:1 n9c**	37.53	44.51	42.07	36.14	6.726	0.7998
**C18:3 n6**	0.11^b^	0.06^b^	0.15^b^	0.35^a^	0.052	0.0089
**C18:3 n3**	0.25	0.19	0.21	0.08	0.073	0.4633
**C21:0**	0.04^c^	0.08^b,c^	0.21^a,b^	0.28^a^	0.041	0.0054
**C23:0**	4.10	4.73	5.28	7.55	1.096	0.1882
**C22:6 n3**	1.70	1.91	1.53	2.08	0.563	0.9054
**ΣSFA**	54.69	47.55	49.68	52.34	5.106	0.7752
**ΣMUFA**	39.12	45.48	42.95	37.32	6.795	0.8288
**ΣPUFA**	2.06	2.16	1.88	2.52	0.682	0.9253
**SFA/USFA**	3.48	1.03	1.11	1.32	1.306	0.5151
**PUFA/MUFA**	1.13	0.05	0.04	0.07	0.555	0.4538
**SFA/PUFA**	57.2^a^	22.6^b^	26.5^b^	20.8^b^	9.039	0.0464
**SFA/MUFA**	16.09	1.09	1.16	1.41	7.601	0.4440
**n-3**	1.95	2.10	1.73	2.17	0.634	0.9633
**n-6**	0.11^b^	0.06^b^	0.15^b^	0.35^a^	0.052	0.0089
**n-3/n-6**	20.1^a^	24.3^a^	13.0^a,b^	6.2^b^	3.119	0.0379
**n-6/n-3**	0.03^c^	0.02^c^	0.09^b^	0.16^a^	0.014	<0.0001

a,b Means within the same row with different superscripts differ significantly (*P* < 0.05).

#### Fatty liver

Table 21 shows the fatty liver incidence in laying hens fed with a reduced methionine and cysteine diet. The results indicated that lower percentage of the fatty liver incidence was found in hens fed with choline propionate at 0.75 kg/t (T7) and 1.0 kg/t (T8) compared to the Control (T5) and the choline chloride group (T6).

**Table 21.** Incidence of fatty liver in laying hens fed with choline product supplementation in Trial 2

Item	Treatment			
	T5	T6	T7	T8
**No. of chicken observed**	108	108	108	108
**Rate for the fatty liver incidence (%)**	30.56	22.22	21.30	21.30

The occurrence of fatty liver lesions in laying hens of treatment groups T5–T8 is presented in Table 22. It was observed that the micro-vesicular steatosis incidence of the liver was lower in the treatment group fed choline propionate product at 0.75 kg/t (T7) and 1.0 kg/t (T8) when compared to the Control (T5) and choline propionate (T6).

**Table 22.** Effect of choline product supplementation on fatty liver lesion severity in laying hens using histopathology scoring with H&E staining in Trial 2

Lesions	Treatment				
	T5	T6	T7	T8	*P*-value
***Liver***					
**Microvesicular**	1 (2-1)	1 (2-1)	1 (1.75-0)	0 (1-0)	0.06
**Macrovesicular steatosis**	0.5 (1.75-0)	1 (1-0)	0 (1-0)	0 (1-0)	0.70
**Hypertrophy**	0 (1-0)	0 (1-0)	0 (0-0)	0 (0-0)	0.11
**Ballooning**	0 (0-0)	0 (0-0)	0 (0-0)	0 (0-0)	–
**Fibrosis**	0 (0-0)	0 (0-0)	0 (0-0)	0 (0-0)	–
***Inflammation***					
**Number of inflammatory foci**	0 (0-0)	0 (1-0)	0 (1-0)	1 (1-0)	0.08
**Microgranulomas**	0 (0-0)	0 (0-0)	0 (0-0)	0 (0-0)	–
**Large lipogranuloma**	0 (0-0)	0 (0-0)	0 (0-0)	0 (0-0)	–
**Portal inflammation**	0 (0-0)	0 (1-0)	0 (1-0)	1 (1-0)	0.08

**Figure 2.**
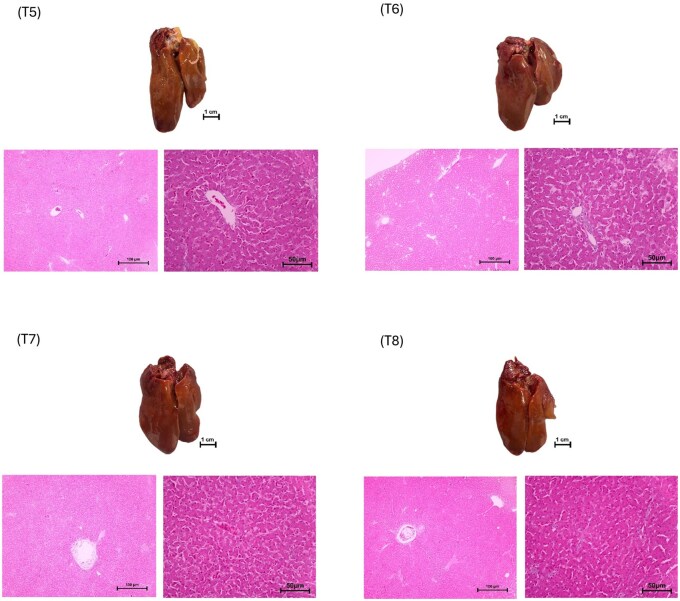
Microscope images illustrating the typical features of liver hepatocytes in terms of gross appearance and sections stained with hematoxylin and eosin (H&E) in laying hens in Trial 2. Pictures in T5, T6, T7, and T8 did not show obvious fatty liver incidence.

From the histopathology observations provided in Figure 2, liver samples from the Control group (T5) and choline-treated groups (T6, T7, T8) did not show any sign of fatty liver incidence.

## Discussion

In the present study, the supplementation of the novel choline propionate product in the hen’s diets resulted in a significant enhancement in both egg production and egg mass. This improvement was observed when the diets were supplemented with choline at application levels of 0.75 and 1.0 kg/t. The addition of choline supplement resulted in an increase in the level of ADFI, egg production and improvement in egg production FCR. Prior studies have revealed that choline plays a significant role in layer diets by improving feed consumption, egg mass, egg yolk weight, and egg production (Parsons and Leeper, 1984; Uzu et al., 1993; Zhai et al., 2013). Studies have shown that supplementing the diet of laying hens with choline and lysine has the potential to improve the immune system and digestive tract health (Kidd, 2004; Vaezi et al., 2011; Abd El-Ghany and Babazadeh, 2022). Based on these observations, we hypothesized that the anion propionate of choline used in this experiment could have enhanced the bioavailability of choline in the birds compared to the inorganic choline chloride treatment group.

The study conducted by Yonke and Cherian (2019), demonstrated that adding 0.1% choline to the diets of layer hens contained in microalgae resulted in an increase in egg production. Choline, an important nutrient in the diets of laying hens acts as a methyl donor and plays a role in the production of phospholipids and the transport of lipids to the egg yolk. However, the study found that choline supplementation had only minor influence on the other egg quality measures that were examined.

Our study is in alignment with a previous trial conducted by Saeed et al. (2017) which revealed that the addition of choline to the diet of laying hens resulted in a decrease in the total lipid content in the liver. Moreover, supplementing the diet with additional choline may reduce the possibility of developing fatty liver and provides advantages to laying hens that could develop fatty liver hemorrhage syndrome (Wolford and Polin, 1975). While another study (Daghir et al., 1960), found no significant impact of choline on the levels of egg yolk and serum cholesterol in laying hens, (Tsiagbe et al., 1988) showed that administering choline at doses of 500 or 1000 mg/kg resulted in significant increases in the concentration of phosphatidylcholine and total phospholipids in egg yolks.

Choline is a recognized lipotropic agent that facilitates the movement of fat stored in the liver, in the form of lipoproteins, to tissues outside the liver where it can be either metabolized or stored (Kettunen et al., 2001; Quarantelli et al., 2007). Consequently, in an optimal condition, the surplus energy from fat is directed towards the formation of muscle protein instead of the production of body fat. This leads to enhanced growth, improved feed conversion ratio (FCR), and reduced lipid content in the liver, abdomen, and carcass (Wen et al., 2014). The decrease in lipid content in liver seen in this investigation is consistent with the findings of (Bhagwat et al., 2024), who showed a reduction in fat content in the abdomen and liver of broilers supplemented with a herbal source of choline.

The liver, a vital organ involved in avian metabolism, is prone to changes affecting its function due to variations in feed contents, mycotoxins and health status of the birds which could be further impacted by stress and environmental variability. The assessment of liver health and functionality involves measuring the activity of serum AST enzymes. These enzymes are released into the bloodstream when liver cells are damaged due to metabolic pressure and hypertension (Corduk et al., 2007). The present investigation pointed to possible hepatic and cellular oxidative stress with elevated serum AST enzyme activity and histological abnormalities in birds that were fed a choline-deficient diet. Notably, this observation was reversed in birds that were supplemented with choline propionate and choline chloride. These findings suggest protective role of the new choline propionate in maintaining liver health. According to a previous report (Khosravinia et al., 2015), choline chloride supplementation has been found to reduce liver damage, as indicated by lower levels of AST in the blood, which is consistent with our data.

In this study, choline propionate supplementation in Trial 1 demonstrated hepatoprotective effects comparable to those observed with choline chloride as shown in the reduced liver lesion scoring and absence of fatty liver morphology when compared to the Control group. The methionine-reduced diet used in Trial 2 did not appear sufficient to induce notable hepatic lipidosis, as evidenced by the absence of significant differences in liver morphology among all the treatment groups.

The rise in the consumption of high in calories and fatty processed food products, containing trans and saturated fatty acids (SFA), has led to a higher prevalence of chronic diseases including obesity, atherosclerosis, hypertension, cardiovascular diseases, type 2 diabetes, and certain types of cancer (Morenga and Montez, 2017). An optimal diet is characterized by a reasonable amount of fat and a high concentration of polyunsaturated fatty acids (PUFA), particularly n−3 PUFA, while minimizing the intake of n−6 PUFA. Egg, a minimally processed food rich in nutrients has one of the greatest concentrations of essential nutrients such as proteins and moderate levels of PUFA, thus provide good nutritional source.

For both Trials 1 and 2, the hens in groups treated with choline chloride supplement resulted in the highest concentration of docosahexaenoic acid (DHA; C22:6 n3) in the eggs, which is an omega-3 fatty acid. In another study, (Wang et al., 2019), it was found that chickens fed diets containing 1,000 mg of choline from microalgae showed a notable increase in the amount of DHA in their egg yolks. In addition, choline acts as lipotropic agents and is involved in the synthesis of phosphatidylcholine from phosphatidylethanolamine N-methyltransferase (Ridgway and Vance, 1992), as well as in the transport of lipids through very-low-density lipoprotein (VLDL) into the egg yolk. The presence of phosphatidylcholine, the primary phospholipid in eggs, may have a biological function given the evident rise in egg weight and egg mass observed in eggs supplemented with choline. Notably, hens supplemented with the novel choline propionate formulation at 0.75 kg/t (T3) exhibited the lowest concentration of saturated fatty acids (ΣSFA), particularly palmitic acid (C16:0), compared to all other groups, including the choline-deficient Control. Given that high dietary intake of saturated fats has been linked to increased plasma low density lipoprotein (LDL)-cholesterol and cardiovascular disease (Micha and Mozaffarian, 2010; Astrup et al., 2020), the observed reduction in ΣSFA in T3 eggs suggests a potential functional health benefit for human consumption. Moreover, total PUFA content (ΣPUFA) was highest in the T3 group, surpassing both the Control and the T2 (choline chloride) group, indicating that choline propionate may enhance hepatic lipid metabolism toward PUFA synthesis or deposition. The ratio of omega-3 to omega-6 fatty acids (n-3/n-6) is another critical marker of egg quality and human health, with optimal ratios associated with reduced inflammatory potential and improved cardiovascular health (Simopoulos, 2016). In the current study, both T2 and T4 groups achieved favorable n-3/n-6 ratios (>6.25), substantially higher than the control (4.24), suggesting that balanced choline supplementation (either chloride or propionate) facilitates a healthier PUFA profile in eggs. Furthermore, the SFA/PUFA ratio, a key indicator of lipid quality, was most favorable in T3 (2.41), lower than both the Control and choline chloride groups. High SFA/PUFA ratios have been associated with increased atherogenic risk, while lower ratios signify improved lipid profiles and reduced cardiovascular disease risk (Hart et al., 2025). Thus, T3 eggs may offer a nutritional advantage for consumers seeking eggs enriched in unsaturated fats while minimizing SFA intake. Taken together, these results suggest that choline propionate not only maintains hepatic lipid mobilization and prevents fatty liver in hens but also delivers favorable fatty acid composition in eggs.

It would be interesting to further investigate the impact of choline chloride and choline propionate on fatty acid profile in eggs especially in markets where higher value functional eggs are appreciated by consumers.

Methionine is the first limiting amino acid in hens fed corn-soybean meal-based diets and as an essential amino acid, it plays a key role in protein synthesis. Methionine can also serve as a methyl donor affecting choline and phosphatidylcholine synthesis, the major phospholipid in egg. In our Trial 2 study, it was found that reducing the levels of methionine and cysteine by 15% in the diets did not have any appreciable impact on the overall productive performance and egg quality of laying hens. Nevertheless, some trend was observed on egg production in which treatment groups supplemented with choline chloride or choline propionate showed numerical improvement. This could be possibly be an indication of methionine sparing effect by choline chloride and choline propionate. The Trial 2 diet did not cause significant negative physiological change to the hens and thus the methionine sparing effect of choline propionate in this study showed a moderate impact. In broiler diets with limited sulfur amino acid content, choline has been shown to partially compensate for methionine deficiency in supporting growth performance, and similarly, methionine can offset low dietary choline levels. As a result, the choline requirement in poultry is influenced by the availability of sulfur-containing amino acids, as well as other methyl donors like betaine and folic acid (Pesti et al., 1981; Baker et al., 1983). Our current study is consistent with previous research which demonstrated that supplementing choline in laying hen diets can enhance egg production and increase egg weight, particularly under conditions where methionine or choline is limited (Beheshti Moghadam et al., 2021).

Although this study did not directly compare choline propionate with herbal or plant-derived choline alternatives, several studies have demonstrated that such products can improve productivity and liver function in poultry. Polyherbal choline has been found to be a feasible alternative to choline chloride in a broiler study where the increasing levels of polyherbal choline led to improved growth performance, enhanced liver color, and reduced hepatic fat content (Taschetto et al., 2025). A trial by Petrolli et al. (2021) supports that plant‑derived choline sources could potentially replace synthetic choline under certain conditions which found improvements in performance metrics without compromising carcass traits (Petrolli et al., 2021). Another study demonstrated that certain plant-based choline with betaine combinations led to higher body weight and more efficient feed conversion ratio (FCR) compared to control and choline chloride groups (Avi et al., 2025). Supplementation with herbal choline sources in broiler diets has demonstrated potential to improve growth performance and liver protection, with some studies reporting comparable or superior effects to synthetic choline chloride on carcass characteristics (Khose et al., 2019). However, due to the inherent variability in plant composition and bioavailability, results may often be inconsistent. Compared to these, choline propionate offers a chemically defined and stable structure with predictable efficacy, warranting further comparative trials to validate its relative performance.

Choline propionate, as an ionic liquid analogue—leverages known mechanisms of improved membrane fluidization, charge delocalization, and micelle or nano-assembly formation to facilitate uptake (Restolho et al., 2012). These properties suggest that choline propionate may act not only as a methyl donor but also as a bioavailability enhancer due to its inherent physicochemical behavior. Further in vitro and pharmacokinetic investigations are warranted to explore its interaction with transporters, its systemic distribution, and its potential role in modulating lipid metabolism at the cellular level.

## Conclusion

This study is the first to demonstrate that dietary supplementation with choline propionate—a novel organic choline source—significantly enhances egg production, egg quality, and liver health in laying hens. Notably, choline propionate not only matched but in some cases surpassed the efficacy of choline chloride in reducing fatty liver incidence at equivalent dosages. Its mild methionine-sparing effect and comparable performance to choline chloride further underscore its functional relevance in poultry nutrition. Choline propionate offers a practical, scalable alternative to choline chloride, particularly valuable in markets where low chloride or chloride-free formulations are preferred due to regulatory or dietary concerns. Its enhanced bioavailability and dual function as lipid metabolism modulator allow for practical effective inclusion rates, without compromising performance, supporting its real-world applicability. Moreover, its liquid form is compatible with existing feed production processes making adoption feasible with minimal formulation or equipment changes. These findings suggest that choline propionate is a viable alternative to choline chloride. The consistent benefits observed across the trials raise interesting questions about its mode of action and metabolic advantages. Future in vitro and mechanistic studies are warranted to elucidate the cellular pathways influenced by choline propionate to unlock its potential as a next-generation feed additive in precision poultry nutrition.

## Abbreviations:

ADFI: average daily feed intake
AST: aspartate aminotransferase
FCR: feed conversion ratio for egg production
H&E: hematoxylin and eosin
Met: methionine
MUFA: monounsaturated fatty acids, corresponding to C16:1n-7, and C18:1n-9
PUFA: polyunsaturated fatty acids, corresponding to C18:2n-6, C18:3n-6, C18:3n-3, and C22:6n-3
SBM-DDGS: soy bean meal with distillers dried grains with solubles
SFA: saturated fatty acids, corresponding to C14:0, C16:0, and C18:0
